# A comprehensive analysis of spermatozoal RNA elements in idiopathic infertile males undergoing fertility treatment

**DOI:** 10.1038/s41598-024-60586-6

**Published:** 2024-05-05

**Authors:** Matthew Hamilton, Stewart Russell, Grace M. Swanson, Stephen A. Krawetz, Karen Menezes, Sergey I. Moskovtsev, Clifford Librach

**Affiliations:** 1 CReATe Fertility Centre, Toronto, ON Canada; 2 Department of Obstetrics and Gynecology, Center for Molecular Medicine & Genetics, C.S. Mott Center, Wayne State University School of Medicine, Detroit, USA; 3 Department of Laboratory Medicine and Pathobiology, University of Toronto, Toronto, ON Canada; 4 Department of Obstetrics and Gynecology, University of Toronto, Toronto, ON Canada; 5 Department of Physiology and Institute of Medical Sciences, University of Toronto, Toronto, ON Canada; 6 Sunnybrook Research Institute, Toronto, ON Canada

**Keywords:** Embryogenesis, Embryology, Gonads, Germline development, Spermatogenesis

## Abstract

Current approaches to diagnosing male infertility inadequately assess the complexity of the male gamete. Beyond the paternal haploid genome, spermatozoa also deliver coding and non-coding RNAs to the oocyte. While sperm-borne RNAs have demonstrated potential involvement in embryo development, the underlying mechanisms remain unclear. In this study, 47 sperm samples from normozoospermic males undergoing fertility treatment using donor oocytes were sequenced and analyzed to evaluate associations between sperm RNA elements (exon-sized sequences) and blastocyst progression. A total of 366 RNA elements (REs) were significantly associated with blastocyst rate (padj < 0.05), some of which were linked to genes related to critical developmental processes, including mitotic spindle formation and both ectoderm and mesoderm specification. Of note, 27 RE-associated RNAs are predicted targets of our previously reported list of developmentally significant miRNAs. Inverse RE-miRNA expression patterns were consistent with miRNA-mediated down-regulation. This study provides a comprehensive set of REs which differ by the patient’s ability to produce blastocysts. This knowledge can be leveraged to improve clinical screening of male infertility and ultimately reduce time to pregnancy.

## Introduction

Infertility affects an estimated 186 million people globally and has considerable psychological, emotional and economic consequences for individuals and couples struggling to conceive and undergoing fertility treatment^1,2^. While male factors contribute to half of all instances of subfertility, current strategies for diagnosing and managing the infertile male are insufficient^3,4^. Spermatozoal quantity, motility and morphology are assessed through laboratory semen analyses^5,6^. In some cases, the workup may include evaluating general and reproductive health through blood hormone testing, reproductive history and physical examination^5,6^. However, 30–50% of infertile males are classified as idiopathic, having an uncertain cause of subfertility^7^.

The complexity of spermatozoa and their potential contributions to conception and offspring development are becoming better understood^3,8^. Beyond carrying the paternal haploid genome, sperm also contain large and small, coding and non-coding RNAs, as well as other epigenetic signatures and proteins^9^. Since mature sperm have a limited cytoplasm, a condensed nucleus, and are largely transcriptionally and translationally inert, these various elements were previously assumed to lack functions beyond spermatogenesis^10,11^. However, accumulating evidence suggests that, upon delivery to the oocyte, they may have important roles in development of the early embryo^12^. For instance, animal studies interfering with sperm RNA activity through gene knockdown, RNase treatment, or using otherwise RNA-deficient sperm have suggested they are indispensable to normal fertilization, embryo development and live birth outcome^13–15^. It has also been demonstrated that the sperm RNA profile may undergo dramatic changes as sperm migrate through the male reproductive tract, specifically throughout epididymal transit, suggesting that particular RNAs may be selectively delivered to sperm through epididymosomes prior to fertilization and are responsive to the environment^16–18^. Interestingly, sperm RNAs, including miRNAs and lncRNAs, may be altered by paternal states such as chronic stress and anxiety, and they provide a potential mechanism for paternally-mediated inheritance of affective behaviour^19,20^. However, while selective RNA delivery during epididymal transit is one proposed mechanism of sperm epigenetic modulation, the dynamicity of the sperm RNA profile in response to paternal influences is not fully understood.

Clinically, sperm RNAs have been reported to be associated with male fertility status. Jodar et al. identified 648 sperm RNA elements that are common among fertile couples^21^. When all of these sequences were present in sperm from idiopathic infertile couples, they were significantly more likely to achieve live birth outcomes by timed intercourse or intrauterine insemination, compared to couples lacking one or more of these species^21^. Recently, our group compared the small RNA profiles of male patients utilizing donor oocytes, who had variable outcomes through assisted reproductive technology (ART), despite normozoospermic presentations^22^. We observed several hundred sperm-borne small RNAs, including 93 micro-RNAs (miRNAs), whose dysregulated expression were associated with blastocyst rates. These data suggest that sperm small RNAs delivered to presumably competent donor oocytes could contribute directly to the embryo’s ability to complete preimplantation development^22^. However, this focused small RNA capture and analysis excludes larger RNA species, which comprise the majority of the RNA elements previously reported by Jodar et al., and further analyzed by Burl et al^21,23^.

In the present study, next-generation sequencing and RNA-seq analysis tools (REDa [RNA Element Discovery algorithm] and TII [Transcript Integrity Index]) were used to evaluate associations between sperm RNA elements and blastocyst progression among normozoospermic males using donor oocytes. We report 33 and 333 RNA Elements (REs), which had increased abundance in sperm samples from couples with high and low blastocyst rates, respectively, some of which were linked to genes related to important developmental processes, including mitotic spindle formation, as well as ectoderm and mesoderm development. We identified 27 RE-associated RNAs (RE-RNAs) that are targets of our previously reported miRNAs which were associated with successful embryo development to the blastocyst and we observed their corresponding target gene depletion consistent with miRNA-mediated down-regulation.

## Methods

Methods related to the selection of study subjects, sample preparation and RNA extraction have been reported in our previous study, in which small RNAs were isolated and profiled from an overlapping patient population at our centre^22^.

### Patient population

Approval for this study was provided by Veritas IRB (Quebec, CA; IRB protocol number 2021–2343- 7435–1). All methods, data collections, and analyses were performed in accordance with the Tri-Council Policy Statement: Ethical Conduct for Research Involving Humans (TCPS 2; 2022) and the Personal Health Information Protection Act (PHIPA; 2004). All participants gave informed consent for the collection and use of their semen and information for research. A total of 47 male patients undergoing IVF using donor oocytes at CReATe Fertility Centre, Toronto, Canada were included in the study. All included patients had an unremarkable semen analysis (normozoospermic; no oligozoospermia, asthenozoospermia or teratozoospermia) and had no other known cause of subfertility. Donor ova were fertilized using either a single sperm provider or sperm from each member of a same-sex male couple (50/50 IVF). Same-sex couples (16 males) were separated into two groups (high and low), based on their blastulation rates. Blastocyst rates were determined through dividing the number of resulting blastocysts by the number of 2PN zygotes obtained. Only couples with a difference in blastulation rate greater than 35% and a minimum of 4 ova fertilized by each partner were included. Single sperm providers (31 males) were subsequently divided into low, average, and high groups based on their relationship to the mean blastulation rate for all male-male couples in the study; cut-offs of one standard deviation higher and lower than the mean were used for the high and low groups, respectively. A minimum of 3 fertilized ova was used for single sperm providers. Patient characteristics and relevant IVF cycle data are summarized in Table 1. Embryology data for individual study subjects is included in Supplementary Table 1. Note that donor age data for 4 patients was unavailable from the biobank database.

**Table 1:** Patient characteristics.

Variable	Low blastocyst rate	Average blastocyst rate	High blastocyst rate	*p*-val*
Sample size (n)	15	18	14	**–**
Sperm provider age (y)	43 (34–57)	42 (34–57)	41 (22–54)	0.7778
Ejaculate volume (mL)	2.7 (0.5–5)	4.0 (1.0–7.2)	3.5 (1.2–5.5)	0.0522
Sperm concentration (millions/mL)	94 (21–253)	85 (21.6–325)	56 (13.6–156)	0.2962
Sperm motility (%)**	48 (32–65)	53 (12–81)	51 (16–72)	0.6548
Sperm morphology (%)***	44 (11–77.5)	41 (15–72.5)	48 (10–67.5)	0.8762
Oocyte donor age (years)	26 (21–34)	27 (21–35)	26 (21–30)	0.7115
No. oocytes retrieved	20 (6–35)	21 (9–36)	20 (3–62)	0.7458
No. mature oocytes retrieved	13 (3–22)	16 (5–30)	15 (3–44)	0.3371
No. 2PN zygotes	8 (2–17)	11 (3–18)	12 (3–40)	0.1650
No. blastocysts	2 (0–3)	6 (2–12)	10 (3–28)	**0.0003**
Blastocyst rate (%)	22 (0–38)	57 (40–67)	83 (70–100)	** < 0.0001**

Age, semen parameters and IVF cycle data are displayed for each study group. *p-values were obtained by applying a Welch’s one-way ANOVA test; significant differences (< 0.05) are bolded. **Total ejaculate motility was used, as opposed to progressive. ***Morphology values reflect the percentage of normal forms identified. A small subset of samples had one value outside of normal ranges published by WHO (reflected in group ranges), but these patients were diagnosed as normozoospermic based on screening semen analyses.

### Sample preparation

Fresh human semen samples were collected by ejaculation. Samples were incubated at room temperature for no more than 60 min and were assessed for: (1) motility and morphology by computer-aided semen analysis (CASA) using the HTM-CEROS Sperm Analyzer (Hamilton Thorne); and (2) morphology according to WHO recommendations^24^. Patient information and samples were collected by members of the CReATe Biobank, a certified CTRNet Biobank Program. Spermatozoa was isolated from seminal plasma 24 h after washing; semen was centrifuged at 420 g for 10 min and the spermatozoal pellet was resuspended in 0.5 mL of sperm wash medium and stored at − 80 °C.

### RNA isolation

RNA extraction procedures were adapted from an existing sperm RNA isolation protocol, described previously^22,25^. Sperm counts were estimated using the CountessTM 3 Automated Cell Counter (Invitrogen) and a starting input of 2 million sperm was used for RNA extraction. Total RNA was isolated using the QIAzol phenol/guanidine-based RNeasy Kit (Qiagen), according to the manufacturer’s instructions. Elution of purified RNA was performed using nuclease-free water. Samples were treated with DNAse I (Thermo Scientific) to remove any single- or double-stranded DNA contamination and starting RNA input was evaluated by Qubit™ RNA HS Assay Kit (Invitrogen). The miRNeasy Kit (Qiagen) was further used to isolate the small RNA species which were analyzed previously^22^.

### Library preparation and sequencing

The SeqPlex RNA Amplification Kit (Sigma Aldrich) was used to reverse-transcribe the total RNA and amplify the resulting double-stranded cDNA through primer-mediated amplification, according to manufacturer instructions. Primers were subsequently eliminated, and DNA was purified using the Zymo DNA Clean & Concentrator-5 (Zymo Research). The NEBNext Ultra II DNA Library Prep Kit (New England BioLabs) was used to prepare and amplify barcoded libraries from purified cDNA. Individual large RNA libraries underwent bead-based size-selection with SPRI beads (Beckman Coulter). Final library traces were assessed using the Bioanalyzer 2100 (Agilent technologies). Resulting large RNA libraries had an average length of roughly 270 bp. Sequencing was performed using the NextSeq 550 Sequencing System (Illumina) for library normalization. Pooled large RNA libraries were denatured and diluted, according to the manufacturer’s protocols, and sequenced at 1 × 150 cycles. Samples were re-pooled using NextSeq reads to calculate normalization, size-selected, re-quantified, and the final sequencing data was collected using the NovaSeq 6000 Sequencing System (Illumina). An S2 NovaSeq flow cell was used, with a read length of 100 bp, paired-end, and a target of roughly 85 million reads per sample.

### Bioinformatics and statistical analysis

Sequence processing and analyses were performed as previously described^26,27^. Briefly, reads were first aligned to the telomere-2-telomere (T2T) genome (T2T-CHM13v2.0) followed by processing with the RNA Element Discovery algorithm (REDa), which identifies exon-sized RNA fragments called RNA Elements (REs), originating from both exonic and intronic regions, as well as those 10 KB from a known exon, and those greater than 10 KB from a known exon (novel orphan)^26^. Reads were normalized per kilobase of exon model per million mapped reads (RPKM) to compare expression levels among samples^28^. Welch’s one-way ANOVA was used to test for significant differences in RE and read categories among groups.

Transcript integrity was evaluated using the transcript integrity index (TII) algorithm aligned to the Gencode hg38 version 41 genome, as described previously, to evaluate the quality of RNA present in the sperm samples, despite the high levels of fragmentation that are inherent in sperm^27^. Differences in human genome alignment versions are due to the TII algorithms requirements of running in R version 3.6.0, which inhibits use of the T2T genome. Transcripts were considered intact if the transcript received a TII score greater than 0.5, which indicates at least half of the transcript was covered by a minimum of 5 reads per million (RPM). Samples not meeting TII thresholds were excluded from the analysis. Transcript integrity was also visually confirmed by the UCSC Genome Browser T2T genome track.

Identified REs were filtered to include only those with greater than 1 RPKM in at least a third of the samples to remove those with low abundance. Mfuzz (version 2.54.0) clustering of group median RPKM was undertaken in R, set for 8 clusters with a membership probability cutoff of 0.4. A Kruskal Wallis test in R was used to apply a p-value for each RE that was present in the cluster patterns. Principal component analysis (PCA) of significant REs was undertaken and a plot was generated using Clustvis (https://biit.cs.ut.ee/clustvis/). A singular value decomposition PCA method was used with imputation of missing values. The Pareto scaling approach was used to scale rows by the square root of the standard deviation.

Gene ontology enrichment analysis was completed using the following programs: EnrichR (https://maayanlab.cloud/Enrichr/); GeneMANIA (cytoscape module version 3.5.2); STINGdb (version 11.5); MsigDB (Human MSigDB v2023.1.Hs); and Metascape (v3.5.20230501). For patterns containing multiple clusters, ontology analysis was undertaken using these major patterns, rather than individual clusters. Significant Mfuzz RE-associated RNAs were compared to miRNA target gene expression. TargetScan and miRtarbase were used to determine and catalog target gene names for previously reported miRNAs^22^. Significant REs were cross-referenced with paternally provided RE lists from Estill et al., 2019, as modified by Swanson, GM et al., 2023 (5 × enriched and 2 × enriched in sperm compared to oocyte)^26,30^.

## Results

### Human sperm contain abundant exonic REs

Overall, 4,767,971,832 reads were obtained from NovaSeq sequencing of 44 samples, which was trimmed to 4,711,072,297 reads (average 107 million reads per sample). The mean number of reads did not differ significantly (Welch’s one-way ANOVA) between high, low and average blastocyst groups (75,783,820 vs 77,856,326 vs 73,173,326), indicating sequencing depth was comparable and sufficient. Reads were mapped to several broad categories, the proportions of which were also similar across blastocyst rate groups, summarized in Supplementary Table 2. The most abundant sequences were human or unclassified in all but one sample (LRNA44), which had a top sequence assigned to the Enterobacteriaceae family. The microbes primarily within this bacterial family are Escherichia coli, which is often present in the ejaculate^31^.

RNA discovery through the REDa identified 435,606 REs across all samples. This number was reduced to 61,549 after filtering for abundant sequences (> 2 RPKM). Their genomic distribution is presented in Table 2. Proportions were highly similar across groups**.**

**Table 2:** Classification of filtered REs.

Low blastocyst rate				Average blastocyst rate				High blastocyst rate			
%Exonic	%Intronic	%10 KB Exonic	%Novel Orphan	%Exonic	%Intronic	%10 KB Exonic	%Novel Orphan	%Exonic	%Intronic	%10 KB Exonic	%Novel Orphan
68.34%	16.70%	6.19%	7.01%	67.89%	16.70%	6.19%	7.01%	70.08%	16.69%	6.20%	7.01%

Percentage is determined by the number of REs with an RPKM > 2 in at least 1 sample per group over the total number of REs that remain following initial filtering.

### Sperm RE and RE-RNA expression differs with blastocyst development rate

**Figure 1:**
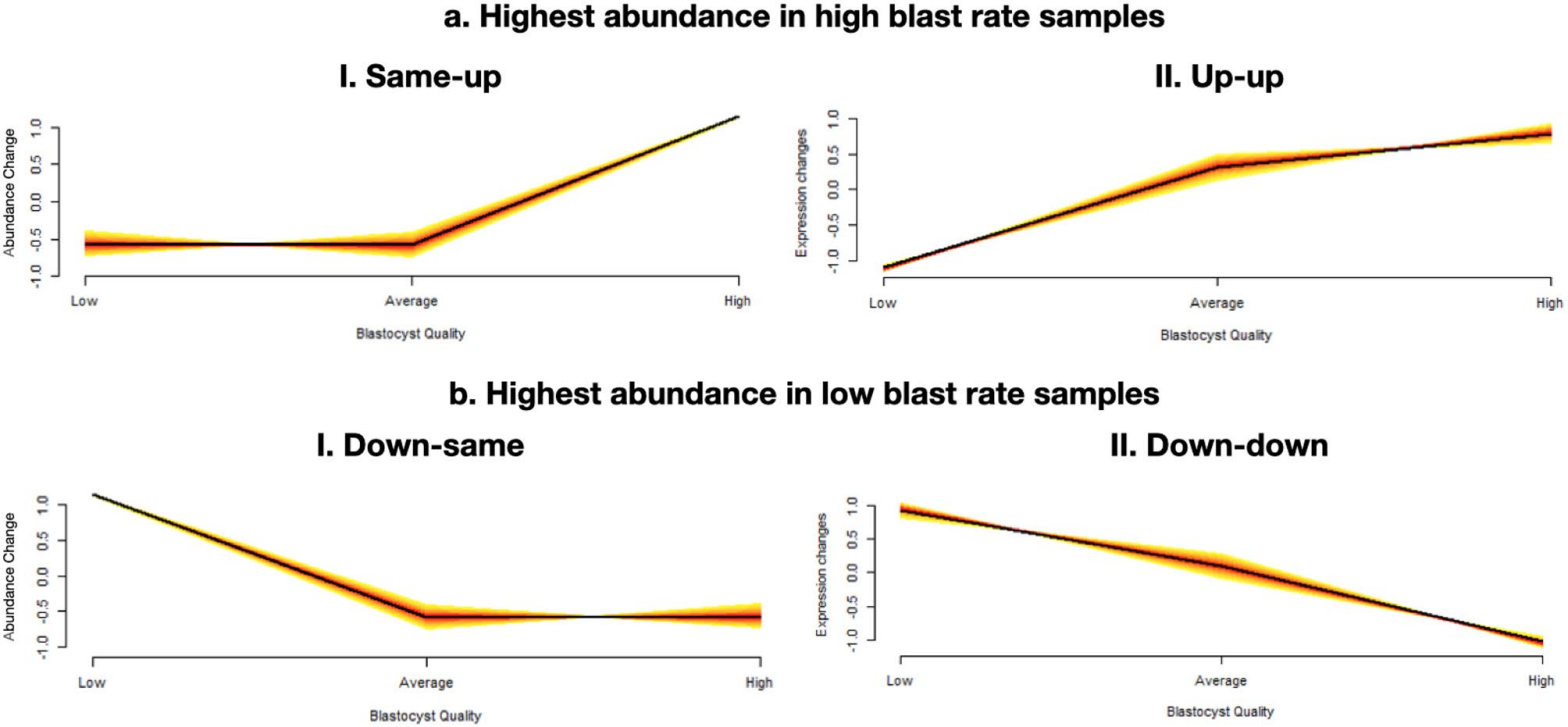
RE clusters related to embryo development. Two major patterns were reflective of REs with highest abundance in the high blast rate group **(a)**: REs moving same-up **(I)** and REs moving up-up **(II)** across groups. Two major patterns demonstrated the highest abundance in low blast rate samples **(b)**: REs moving down-same **(I)** and REs moving down-down **(II)** across groups. Graphs are visualized across blastocyst rate groups based on fold-change in the abundance of the cluster patterns.

Previous reports suggest that sperm-borne REs are associated with male fertility in idiopathic cases. To investigate associations between sperm REs and resulting embryo development rates in a presumably normal population of patients, we leveraged state-of-the-art bioinformatic analyses. Mfuzz clustering identifies patterns of RE expression changes based on the group median RPKM. Based on the number of clusters, alongside required parameters for pattern membership (how closely the RE expression changes follow the pattern), REs are segregated into specific patterns. Here, we identified 8 patterns. A sample-sample membership of 0.4 was used when clustering, indicating REs must closely follow the pattern. A total of six major patterns were identified based on differential abundance across blastocyst rate groups (Supplementary Fig. 1). These patterns are referred to as same-up, up-up, down-same down-down, up-down and down-up, based on how RE abundance changed with increased blastocyst rate. For example, same-up refers to REs following a pattern of similar abundance between low and average groups (same) with increased abundance in the high group (up), while down-down refers to REs following a pattern of highest abundance in the low group with lower abundance in the average group and further decreased abundance in the high group. Of these major patterns, those related to embryo development were comprised of elements with significantly higher abundance in the high blastocyst rate group (same-up and up-up) and elements with significantly higher abundance in the low blastocyst rate group (down-same, down-down), shown in Fig. 1.

**Figure 2:**
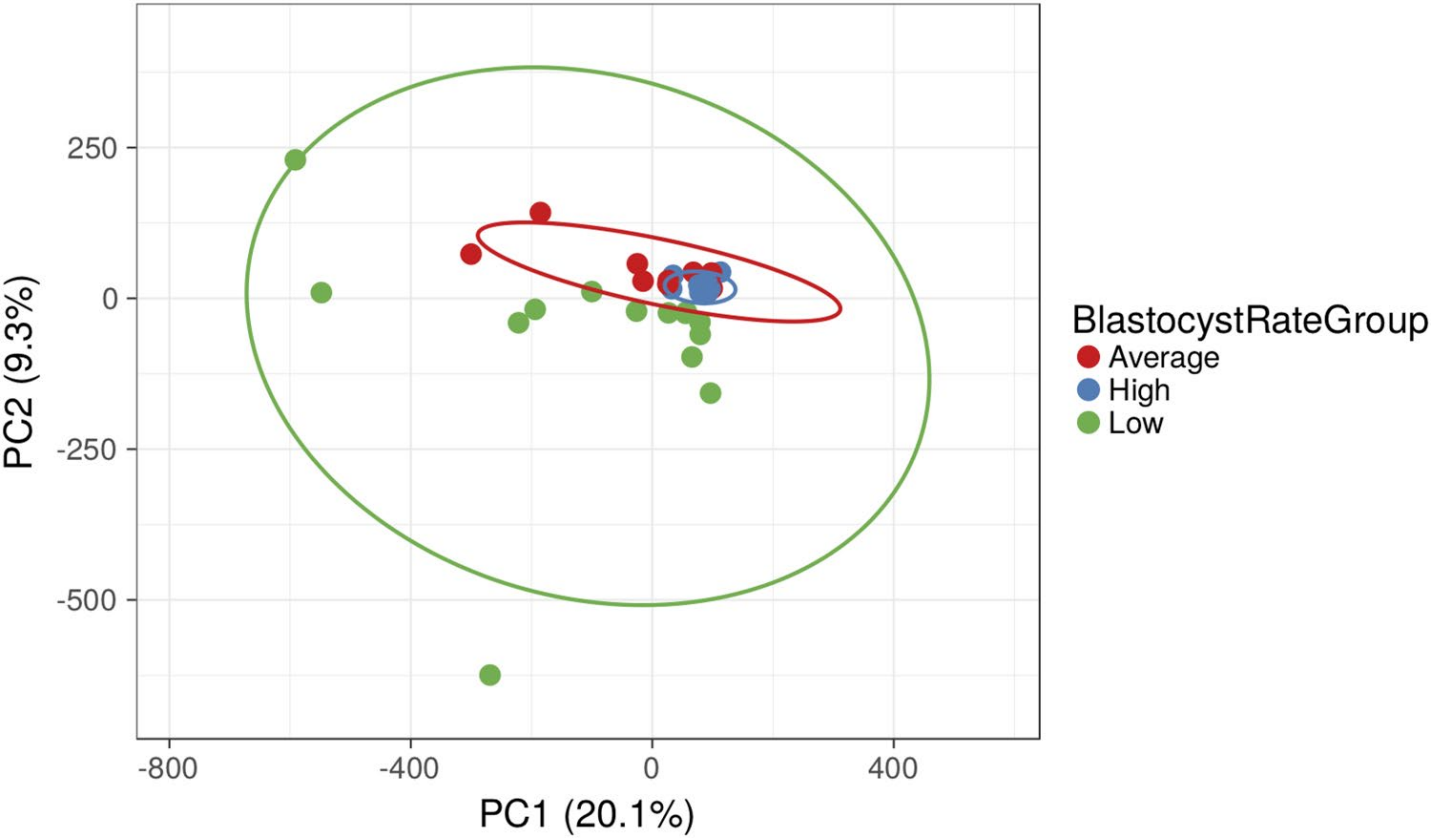
Principal component analysis of significant REs. Each point represents an individual sample and blastocyst rate groups are indicated by color. Variation values of PC1 and PC2 are 20.1 and 9.3%, respectively.

A total of 12,519 REs comprised the four Mfuzz patterns most related to blastocyst development, of which 366 were statistically significant (*padj* < 0.05). Principal component analysis (PCA) of significant REs showed clustering amongst blastocyst rate groups, but more variability with decreasing blastocyst rate (Fig. 2). However, there was overlap between some members of the average with the high group. This suggests that there may be a specific RE signature required to support normal blastocyst development, while variations in the sperm-borne RE complement could reduce blastocyst rates.

The number of REs with significant differences between groups, and the corresponding number of related significant genes are reported in Table 3, with the complete list of significant REs contributing to the major patterns included in Supplementary Table 3.

**Table 3:** REs and significant genes identified in major clustering patterns.

Trend	Pattern	REs	Significant REs (p < 0.05)	Unique gene IDs (*p* < 0.05)	Common gene names (*p* < 0.05)
Increased in high blast rate group	Same-up	1758	31	32	23
	Up-up	754	2	2	1
Increased in low blast rate group	Down-same	8948	304	160	138
	Down-down	1059	29	9	8

Importantly, the down-same pattern reflects 304 significant REs and 138 RE-associated RNAs (RE-RNAs) which are up-regulated in the low group, compared to both the average and high groups. For the up-up pattern, only one common RE-RNA was found to increase with increasing blastocyst rate (from low to average to high): PPP4R1. In contrast, 8 RE-RNAs decreased with increasing blastocyst rate (from high to average to low): *RNA45S1*, *RNA18S1*, *RNA5-8S1*, *RNA28S1*, *ITGAM*, *VPS53*, *COQ8B*, and *Z82195.3*.

### Sperm RE-RNAs are involved in developmentally important processes

A summary of findings from the gene ontology (GO) enrichment analysis is included in Supplementary Table 4. For gene patterns reflecting highest abundance in the high blastocyst rate group, genes moving same-up are involved in cellular processes such as the peroxisomal oxidation and mitosis, while the RE-RNA for the up-up pattern (*PPP4R1*), is related to phosphorylation and dephosphorylation. For patterns reflecting highest abundance in the low blastocyst rate group, RE-RNAs moving down-down are involved in processes related to immune cells (e.g., macrophages, neutrophils, microglial, leukocytes), as well as ectoderm development (*ITGAM*), while RE-RNAs moving down-same are involved in mitotic spindle formation (*DLG1, ACTN4, HDAC6, PIF1*, and *ARHGEF7*) and mesoderm development (*OVOL1, PRKACA*, and *TXNRD1*).

### Sperm RE and RE-RNAs are related to MiRNA enrichment

REs with significant differences between groups of blastocyst development were compared to the differentially expressed miRNAs from overlapping samples as reported previously^22^. Specifically, lists of miRNAs which were enriched in the low and high blastocyst rate groups were searched against miRNAs using TargetScan and the miRTarBase. Overlapping RE-RNA movement for miRNAs enriched in the high and low blastocyst rate groups is summarized in Table 4.

**Table 4:** RE-RNAs overlapping with enriched MiRNAs.

Table descriptor	Number of unique target names	Total number of shared genes (all Mfuzz significant/target genes)	Overlapping RE-RNA names
TargetScan enriched in Low	815	9/815	AP1G1**, FBXO32**,* GSE1****,*** MAPK8**,* NDRG2****,*** PHC3*,* TGFBR3****,*** UGCG**, WNK1**
TargetScan enriched in High	1176	13/1176	AKIRIN1*, ATF2, CIT, DLG1, FBXO32**, FGF10, MAP3K1, MAPK8**, TMEM63B, TNR, UGCG**, USP37*, ZRANB2
miRTarBase enriched in Low	927	9/927	ATM, CS, DAZAP2, EEF2**, MOCS3***,**** NPM1****,*** PHC3*, VPS53***,**** ZNF675*
miRTarBase enriched in High	1100	15/1100	ACTN4, AKIRIN1*, AP1G1**, ATM**, DAZAP2**, EEF2**, FAXC, PRRC2C, PTMA, QKI, RAB13, SYNJ2, TPM3, USP37*, WNK1**

* *RE-RNAs* are in both databases for either ‘enriched in High’ or ‘enriched in Low’ comparisons.

** *RE-RNAs* are in both the ‘enriched in High’ and ‘enriched in Low’ comparisons for at least one database.

**Figure 3:**
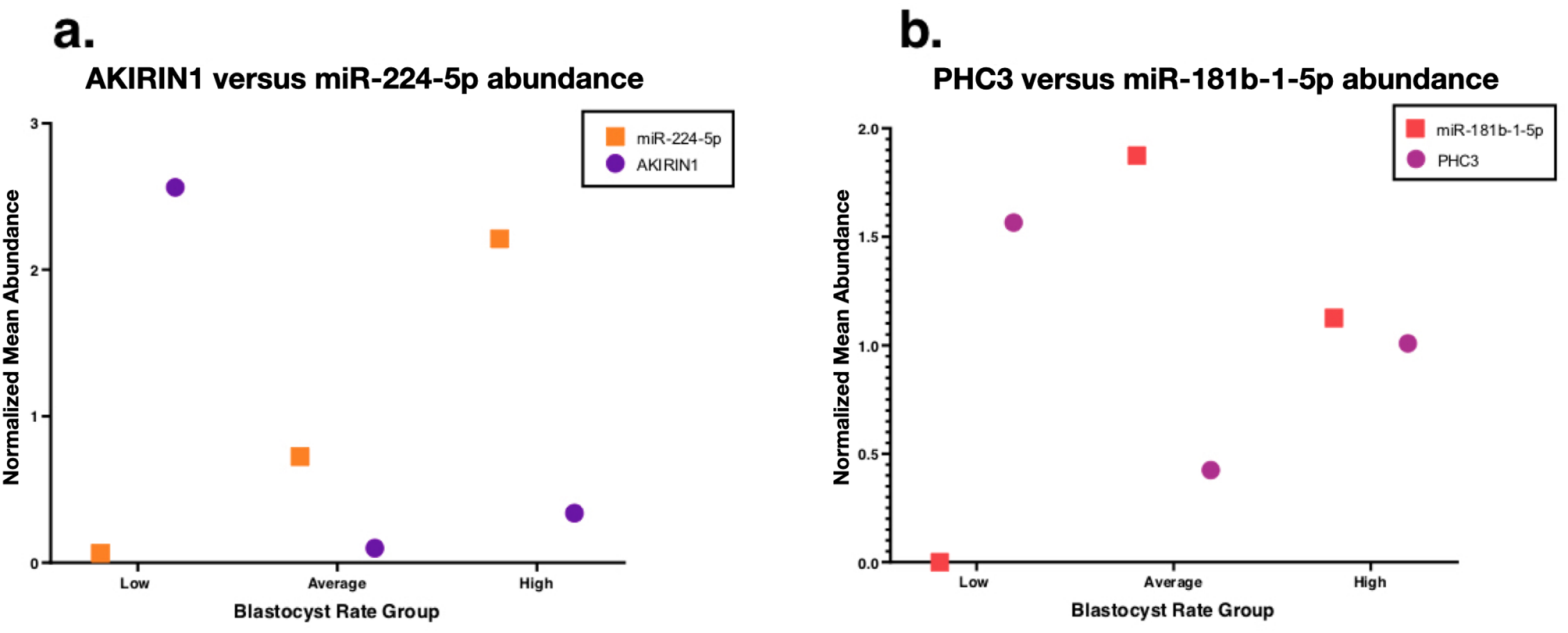
Enrichment of miR-associated RE-RNAs Across blastocyst rate groups. The abundance of *AKIRIN1* (**a**) and *PHC3* (**b**) are plotted against miR-224-5p and miR-181b-1-5p, respectively. A proportion of mean abundance for each blastocyst rate group relative to overall mean abundance (across all samples) was used to normalize abundance between the genes and miRNAs of interest. *AKIRIN1* includes: *AKIRIN1-201*; *AKIRIN1-202*; *AKIRIN1-203*; and *AKIRIN1-204*. *PHC3* includes: *PHC3-215*; *PHC3-214*; and *PHC3-209*.

The *PHC3* and *AKIRIN1* RE-RNAs, which both move down-same in relationship to blastocyst rate groups, were associated with hsa-miR-181b-5p and hsa-miR-224-5p, respectively, across both databases (Fig. 3). *USP37* was associated with hsa-miR-19a-3p in both databases, however this RE-RNA moves up-down in relationship to blastocyst progression, with highest abundance in the average group. A complete list of miRNAs associated with each overlapping gene for individual databases is included in Supplementary Table 5.

Nine RE-RNAs are targets of miRNAs that were enriched in the low blastocyst development group. Interestingly, these 9 RE-RNAs are depleted in these samples, suggestive of miRNA-mediated gene regulation. Further, a corresponding decrease in the significant REs was also noted for 5 of the 9 RE-RNAs (*GSE1, MOCS3, NDRG2, NPM1,* and *ZNF67*5). Moreover, 18 RE-RNAs are targets of miRNAs that were enriched in the high blastocyst rate group. All significant Mfuzz REs were decreased for all 18 RE-RNAs, and 14 of 18 RE-RNAs were depleted in all samples. Of the remaining four RE-RNAs, two are 5’ depleted in all samples (*TMEM63B, USP37*) and two are full-length in at least 1 sample, but are depleted in the remaining samples (*CIT, QKI*). Interestingly, the *TMEM63B* RE is paternally provided according to previously published datasets. Specifically, *TMEM63B* is five-fold enriched in sperm compared to the oocyte^30^. TMEM63B functions to enable calcium-activated cation channels and its knockdown results in preweaning lethality in mice (complete penetrance), suggesting it may have a highly important developmental role^32,33^. *TMEM63B* is targeted by hsa-miR-19a-3p and hsa-miR-145-5p (which were enriched in the high blastocyst rate group). Eleven other blastocyst rate-associated RE-RNAs were identical to those defined previously as paternally-provided REs (Table 5). These RE-RNAs are either five-fold or two-fold enriched in sperm, compared to the oocyte. Two of the 12 RE-RNAs, *TMEM63B* and *CCDC9,* overlap with the paternally-provided datasets. *CCDC9*, which is full-length in two samples, but depleted in the remaining samples, is also five-fold enriched in sperm compared to the oocyte and functions to enable RNA binding^34^.

**Table 5:** Paternally-provided REs and associated genes.

Fold-Enrichment (Estill, MS et al., 2019, Swanson, GM et al., 2023)^24,28^	RE-RNA	Paternally provided RE (hg38)	Mfuzz RE (T2T)
**5 × paternally enriched**	*ADAT1*	chr16_75622717_75622816	chr16_81656516_81656615
			chr16_81657135_81657280
	***CCDC9***	**chr19_47271274_47271953**	chr19_50082866_50083243
			chr19_50083423_50083737
			chr19_50083893_50083997
			chr19_50085922_50086173
			chr19_50089933_50090016
			chr19_50095881_50096016
			**chr19_50096602_50097265**
	*FUS*	chr16_31189665_31189794	chr16_31577637_31578263
	*KIAA0586*	chr14_58487007_58487166	chr14_52665509_52665581
			chr14_52666879_52667106
		chr14_58472199_58472279	chr14_52672869_52673063
			chr14_52674769_52674956
			chr14_52764218_52764317
	*NPIPA8*	chr16_18321017_18321124	chr16_18328260_18328367
			chr16_18339845_18339976
	*NPIPB15*	chr16_74389995_74390030	chr16_80195645_80195806
			chr16_80310038_80310199
			chr16_80437867_80438580
	*SEC31A*	chr4_82818661_82819253	chr4_86172963_86173787
			chr4_86178084_86178257
			chr4_86186231_86186409
			chr4_86204976_86205090
	TMEM63B*	**chr6_44148792_44148945**	**chr6_43982247_43982716**
			chr6_43971829_43971972
			chr6_43972268_43972386
		**chr6_44149859_44149965**	**chr6_43983314_43983420**
			chr6_43973707_43973968
			chr6_43974483_43974553
		chr6_44150224_44150310	chr6_43980302_43980382
			chr6_43980832_43980955
			chr6_43981707_43981840
		chr6_44153676_44153843	chr6_43981968_43982105
			chr6_43988147_43988974
	*ULK4*	chr3_41717728_41717861	chr3_41722772_41722823
			chr3_41732733_41732789
			chr3_41732943_41733064
	*WNK1*	chr12_897479_897681	chr12_854953_855172
			chr12_856734_857064
			chr12_880361_881796
			chr12_904228_907208
**2 × paternally enriched**	PHC3*	chr3_170136419_170136665	chr3_172872255_172882054
		chr3_170145423_170145521	
	*PIAS2*	chr18_46829734_46829867	chr18_47046007_47046097
		chr18_46890580_46891054	chr18_47046227_47046277
			chr18_47054826_47054910

Overlapping REs and their associated RNAs (RE-RNAs) are bolded. Asterisk (*) indicates the gene is a miRNA target listed in Table 4.

## Discussion

Next-generation sequencing was used to profile RNA extracted from the spermatozoa of males with normal semen parameters, but variable IVF outcomes. We identified 61,549 abundant REs across all human sperm samples through the REDa RNA discovery pipeline, which included exonic, intronic and novel uncharacterized REs. A previous sperm RNA-seq study identified 185,037 REs in boar sperm, with the top 10% of these REs accounting for 65% of the read count^35^. The 18,504 REs occupying this top decile followed a similar distribution to the REs presented in this study, with 69% exonic REs and 12% intronic REs (compared to 68 and 17%, respectively), though almost 15% were novel orphan REs (compared to 7%)^33^. While a number of other sperm RNA-seq studies have been conducted in a variety of species, the human sperm transcriptome is comparatively understudied^35–38^. Further validation across species with a specific focus on the human sperm transcriptome would be valuable.

Clustering of REs revealed differences between blastocyst development groups. The low blastocyst rate group had the most unique profile of REs, with 304 REs (and 138 common RE-associated RNAs (RE-RNAs)) significantly more abundant compared to the average and high blastocyst rate groups. While the high blastocyst rate group had the most unique small RNA profile in our previous study, the enrichment of REs reported here in the low blastocyst rate group may reflect differences in small RNA regulation (and specifically, RNA interference), which influence the amount of fully intact REs that are present^22^. Overall small and total RNA variation between groups may be indicative of gene regulation which influences the competence of preimplantation embryos and specifically, their ability to progress to the blastocyst stage. While the mechanisms through which sperm RNA elements may regulate preimplantation embryonic development are unknown, it has been proposed that they may alter retroelement activity upon delivery to the oocyte^17,39^. For instance, targeted transfer RNA-derived small RNA (tsRNA) depletion through co-injection of antisense sequences (along with sperm) into porcine oocytes led to aberrant cleavage and blastocyst progression whilst altering embryonic expression of retroelements^40^. Specifically, L1M3b of LINE-1 family, which was previously shown to regulate chromatin accessibility in murine embryos, was downregulated^40,41^. Interestingly, one unique gene identified here, CHM13_T1000172, which was significantly enriched in the low blastocyst rate group compared to the average and high blastulation groups (down-same pattern), is located on chromosome Y and overlaps with LINE L1 repeat L1PA10. This finding supports the hypothesized involvement of retroelements, either directly or indirectly, in the sperm RNA-mediated modulation of early embryo development.

Gene ontology enrichment analysis of genes associated with the significant REs revealed their involvement in critical developmental processes, including mitotic spindle formation and ectoderm and mesoderm development. These findings suggest that there are developmentally important genes and their associated RNA elements which are differentially represented in the sperm of males with lower rates of blastocyst development, compared to those with higher rates. The association of significant genes with primary germ layer formation supports numerous studies which have suggested that modifying the sperm RNA payload can influence offspring phenotypes beyond blastulation^42–44^. For instance, in a study by Chen et al., not only did mice consuming a high-fat diet demonstrate changes in their RNA payload–specifically tsRNAs–the injection of sperm tsRNAs from mice consuming this diet into normal zygotes altered metabolic gene expression in early embryos and induced metabolic disorders in the offspring^44^. While the precise mechanisms through which paternally-delivered elements may influence development are unclear, regulation of transcriptional and translational machinery in the oocyte likely involves a complex interplay between numerous factors, including transposable elements, DNA methylation and chromatin structure^45^.

Further, comparing significant RE-associated RNAs (RE-RNAs) with miRNA target gene abundance across blastocyst rate groups revealed 9 and 18 RE-RNAs that are targets of miRNAs that were enriched in the low and high blastocyst rate groups, respectively. The majority of these gene targets were depleted or 5’ depleted in all samples and many of the significant REs associated with these genes had corresponding decreases. Depletion of these targets could indicate that these RE-RNAs are silenced as they are no longer required, perhaps beyond sperm maturation. Alternatively, these discoveries could indicate a potential miRNA-mediated downregulation of genes that are differentially expressed among males with variable IVF outcomes. Sperm-delivered miRNAs have previously been linked to sperm characteristics and IVF outcomes^46–48^. Importantly, the specific enriched human miRNAs reported here have also been previously reported to influence developmental processes. In particular, hsa-miR-181b-5p, which was enriched in the sperm of the low blastulation group, has previously been shown to inhibit trophoblast migration and invasion^49^. Interestingly, miR-181b-5p was also previously identified in both mouse spermatozoa and epididymosomes^50^. Hsa-miR-224, which was also enriched in the low blastocyst rate group, has been functionally-related to the differentiation of spermatogonial stem cells in mice^51^. Higher expression of miRNA-19a/b-3p has previously been associated with oligoasthenozoospermia and male infertility^52^. While miRNA-19a/b-3p was indeed down-regulated in the high blastocyst rate group, it was also reduced in the low group, suggesting its activity in normozoospermic males may require further elucidation.

Finally, 12 significant RE-RNAs overlapped with previously published datasets of paternally-provided RNAs, with two of these 12 RE-RNAs as exact overlapping genomic regions between datasets: *TMEM63B* and *CCDC9*. With the exact genomic region overlapping, the significance of these RE-RNAs as paternally-derived is highlighted. Importantly, knockdown of TMEM63B results in preweaning lethality, which suggests it may have a significant role in embryo development^32^. CCDC9 was also previously identified as a novel candidate gene of severe asthenozoospermia and has been reported to regulate sperm motility and spermiogenesis^53,54^. Moreover, CCDC9 has also previously been associated with ZC3H11A, an mRNA binding protein that is required for mouse embryo development to the peri-implantation stage^55^.

This study is limited by a relatively small sample size and the analysis of only one ejaculate from each included patient (to limit our analysis to only samples for which direct embryology outcomes were available) and therefore still needs validation to confirm that specific REs were not identified by random chance. However, while these samples were assumed to be reflective of each patient’s overall semen profile, it should be noted that the RNA sequencing data presented does not capture the heterogeneity that may exist across repeated ejaculates or between individual sperm. Additionally, DNA fragmentation indexes were not available for the population studied as DNA fragmentation is not routinely used at this clinic. Thus, this study is also limited by the significant fragmentation that is inherently present within sperm, which may not only influence blastocyst progression itself, but it also influences the number of intact transcripts which may be analyzed (despite cut-offs for sequence quality). Use of the Transcript Integrity Index (TII) algorithm begins to combat the transcript integrity aspect of this limitation as it was specifically designed for RNA quality assessment of samples exhibiting high RNA fragmentation rates^29^. Using a cut-off optimized in human sperm of a minimum 5 RPM across half the transcript as a measure of transcript integrity, combined with visualization of transcripts on the UCSC Genome browser, enabled identification of full-length RE-RNAs for evaluation. Furthermore, the TII algorithm has the capability of determining whether samples contain similar RNA quality, for downstream analysis. Further study in larger patient populations could corroborate the identified relationship between the presence of the identified significant sperm-borne RNAs and IVF outcomes; including embryo development, and pregnancy and live birth rates. Gene knock-out and other mechanistic studies in sperm and embryos, specifically targeting the RNA elements and genes presented here, could elucidate the functional pathways and genetic underpinnings through which RNAs delivered by sperm may direct blastocyst progression and embryogenesis.

High resolution methods for assessing sperm competence are currently lacking. An RE-based assay for assessing sperm quality, and its ability to generate blastocysts in the absence of female factors, could improve patient counseling and reduce time to pregnancy. With further investigation and clinical validation, modifying important RNAs within sperm or selecting sperm based on their RNA contents could offer novel therapeutic strategies to improve clinical outcomes and ameliorate the burden of infertility.

### Supplementary Information

Supplementary Information 1.

Supplementary Information 2.

Supplementary Information 3.

## Data Availability

The data used in this analysis can be accessed under accession number GSE262969.
