# Supplementary material for: A comprehensive analysis of spermatozoal RNA elements in idiopathic infertile males undergoing fertility treatment
Source: Sci Rep. 2024 May 5;14:10316. doi: 10.1038/s41598-024-60586-6 (PMC11070429; doi:10.1038/s41598-024-60586-6)
Supplement: Supplementary file 1 — Supplementary Information 1. [file 41598_2024_60586_MOESM1_ESM.pdf]

A comprehensive analysis of spermatozoal RNA elements in idiopathic infertile males undergoing fertility treatment

Matthew Hamilton[a], Stewart Russell[a], Grace M Swanson[b], Stephen A Krawetz[b], Karen Menezes[a], Sergey I. Moskovtsev[a,c], and Clifford Librach[a,d,e,f]

a CReATe Fertility Centre, Toronto, Ontario, Canada

b Department of Obstetrics and Gynecology, Center for Molecular Medicine & Genetics, and C.S. Mott Center, Wayne State University School of Medicine, Detroit, USA.

c Department of Laboratory Medicine and Pathobiology, University of Toronto, Toronto, Ontario, Canada

d Department of Obstetrics and Gynecology, University of Toronto, Toronto, Ontario, Canada

e Department of Physiology and Institute of Medical Sciences, University of Toronto, Ontario, Canada

f Sunnybrook Research Institute, Toronto, Ontario, Canada

Supplementary Figure 1: Major clustering patterns identified between blastocyst rate groups

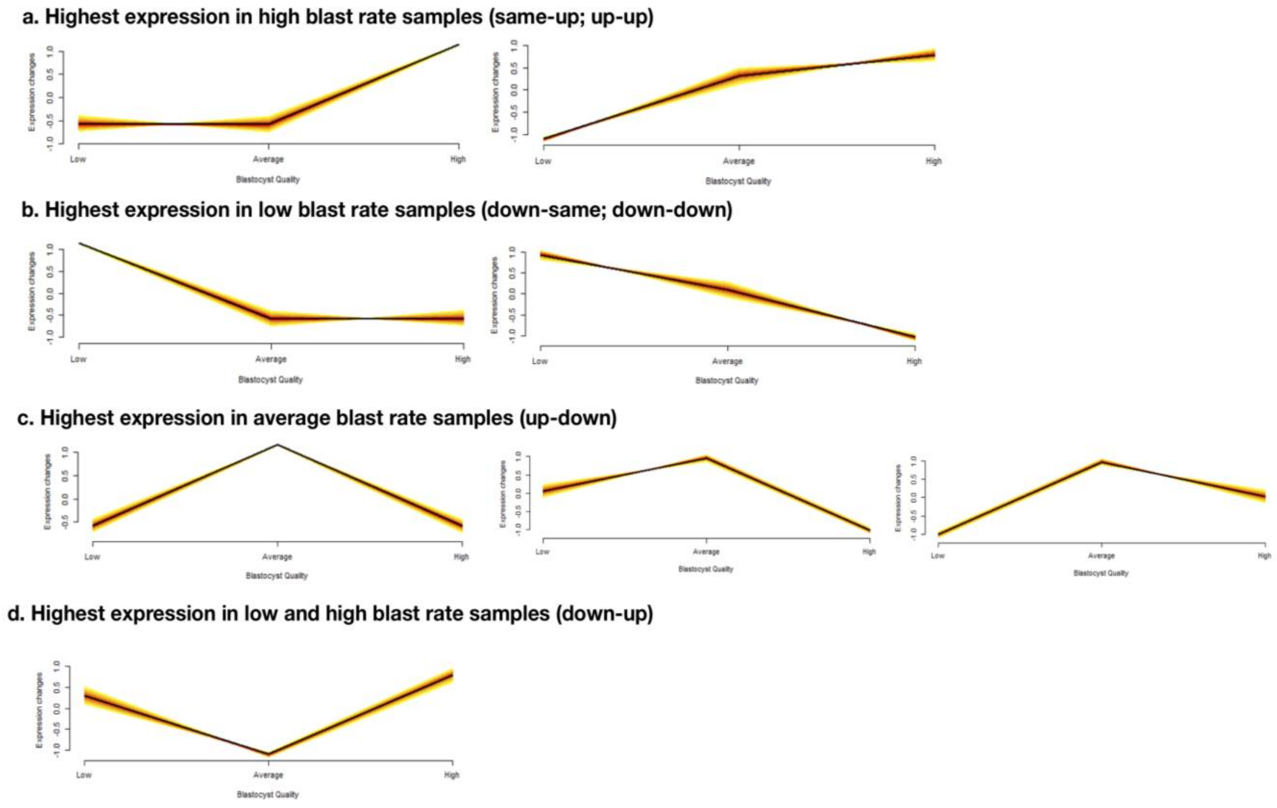

Supplementary Figure 2: Individual Sample Abundance of RE-associated RNAs

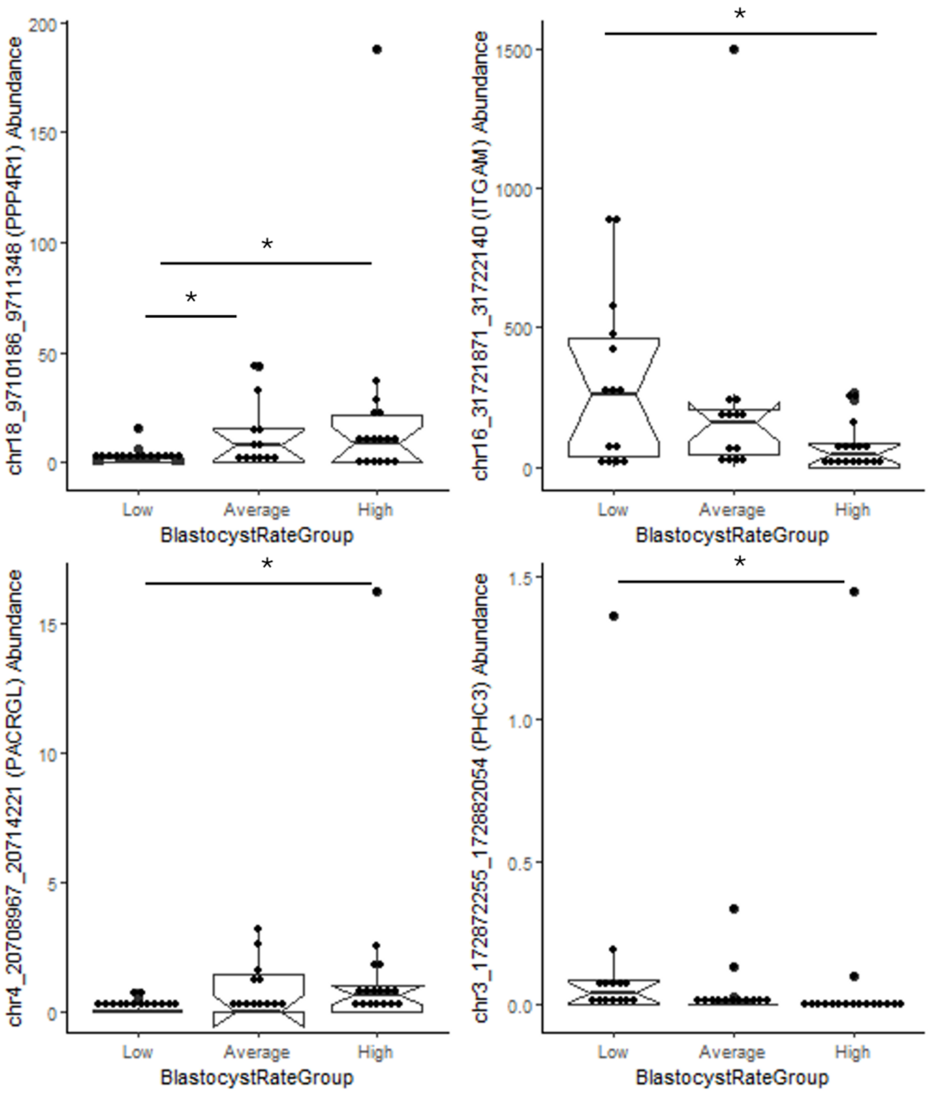

* Wilcoxon Mann-Whitney u-test *p*-value was significant

Supplementary Table 1: Embryology Data for Individual Study Subjects

Sample	Age	Donor Age	No. Oocytes Retrieved	No. MII Oocytes	No. 2PN Zygotes	No. Blastocysts	Blastocyst rate (%)
**LRNA1**	45	28	19	12	6	1	17%
**LRNA6**	52	23	12	7	6	0	0%
**LRNA13**	44	25	6	6	6	2	33%
**LRNA18**	38	29	31	22	7	2	29%
**LRNA19**	54	25	13	11	8	3	38%
**LRNA20**	34	23	35	15	11	4	36%
**LRNA23**	37	27	35	21	17	6	35%
**LRNA24**	47	34	27	15	6	2	33%
**LRNA29**	45	unavailable	6	3	2	0	0%
**LRNA35**	38	34	11	8	8	2	25%
**LRNA37**	39	30	22	9	7	0	0%
**LRNA40**	57	25	16	10	7	1	14%
**LRNA41**	34	23	21	13	13	3	23%
**LRNA48**	44	21	22	17	11	1	9%
**LRNA53**	36	24	20	20	9	3	33%
**LRNA2**	39	28	18	12	12	10	83%
**LRNA7**	42	23	12	7	4	3	75%
**LRNA8**	45	27	12	7	5	4	80%
**LRNA26**	51	33	22	13	12	10	83%
**LRNA28**	54	unavailable	4	3	3	3	100%
**LRNA31**	44	unavailable	3	3	3	3	100%
**LRNA34**	42	unavailable	10	6	5	4	80%
**LRNA38**	31	23	20	12	10	7	70%
**LRNA42**	38	27	62	44	40	28	70%
**LRNA44**	34	26	11	7	5	5	100%
**LRNA45**	44	21	32	29	21	19	90%
**LRNA47**	36	24	21	20	13	10	77%
**LRNA50**	22	21	25	15	12	9	75%
**LRNA51**	51	30	38	33	28	23	82%
**LRNA52**	41	24	20	20	8	6	75%
**LRNA58**	47	24	26	23	17	14	82%
**LRNA59**	42	28	9	8	7	6	86%
**LRNA60**	36	29	14	12	9	8	89%
**LRNA14**	44	25	9	5	3	2	67%
**LRNA16**	44	29	25	21	18	12	67%
**LRNA25**	46	31	30	15	11	7	64%
**LRNA27**	38	23	36	24	12	8	67%
**LRNA30**	57	28	22	18	10	6	60%

**LRNA32**	35	21	13	9	4	2	50%
**LRNA36**	49	35	21	17	10	5	50%
**LRNA39**	34	26	12	8	7	4	57%
**LRNA43**	35	23	19	16	12	8	67%
**LRNA49**	40	24	23	21	18	12	67%
**LRNA54**	37	25	31	30	14	7	50%
**LRNA55**	52	27	21	16	14	7	50%
**LRNA56**	36	31	14	9	4	2	50%
**LRNA57**	42	30	17	15	15	6	40%

Supplementary Table 2: Categorical mapping of sequencing reads

A. Categorical mapping for individual samples

Group	Samples	Adapter_badquality	rRNA (Human)	Mitochondrial (Human)	Genomic (Human)	Bactviral	Repbase	Unmapped
low	1	0.0100	0.0536	0.0523	0.2077	0.3617	0.0011	0.3135
low	6	0.0134	0.1800	0.1188	0.1883	0.3979	0.0014	0.1002
low	13	0.0150	0.1869	0.0237	0.0592	0.2775	0.0004	0.4374
low	18	0.0105	0.0096	0.0082	0.0178	0.2293	0.0000	0.7245
low	19	0.0132	0.1439	0.1495	0.3254	0.2143	0.0025	0.1511
low	20	0.0094	0.0587	0.0136	0.0818	0.4527	0.0002	0.3835
low	23	0.0158	0.0043	0.0030	0.0513	0.2406	0.0003	0.6848
low	24	0.0123	0.0649	0.0402	0.0781	0.3543	0.0004	0.4498
low	35	0.0127	0.0675	0.0475	0.3797	0.3763	0.0042	0.1120
low	37	0.0244	0.0006	0.0001	0.0278	0.4754	0.0003	0.4715
low	40	0.0087	0.0612	0.2040	0.1617	0.2033	0.0006	0.3604
low	41	0.0103	0.0059	0.0030	0.0459	0.3732	0.0001	0.5615
low	48	0.0079	0.1435	0.1004	0.5352	0.1863	0.0018	0.0249
high	2	0.0079	0.1739	0.0682	0.1150	0.2225	0.0006	0.4119
high	7	0.0077	0.0075	0.0101	0.0141	0.3496	0.0002	0.6108
high	8	0.0109	0.0069	0.0019	0.0130	0.6628	0.0000	0.3045
high	26	0.0121	0.0729	0.0446	0.1073	0.3452	0.0006	0.4171
high	31	0.0198	0.0292	0.0212	0.0715	0.0905	0.0002	0.7676
high	34	0.0111	0.0814	0.0866	0.3670	0.3723	0.0018	0.0798
high	38	0.0092	0.0006	0.0000	0.0834	0.3396	0.0002	0.5670
high	42	0.0107	0.0052	0.0033	0.0304	0.1989	0.0001	0.7513
high	44	0.0152	0.0028	0.0017	0.0198	0.6489	0.0002	0.3115
high	45	0.0081	0.3069	0.1002	0.1742	0.3586	0.0003	0.0516
high	47	0.0100	0.1374	0.2480	0.1970	0.2412	0.0006	0.1658
high	51	0.0110	0.0795	0.0509	0.0521	0.2514	0.0003	0.5547
high	52	0.0153	0.0401	0.0174	0.0398	0.3234	0.0003	0.5638
high	60	0.0090	0.0758	0.0362	0.0946	0.3006	0.0004	0.4834
average	14	0.0104	0.1325	0.0419	0.1425	0.2138	0.0010	0.4579
average	16	0.0114	0.0933	0.0221	0.1064	0.2060	0.0007	0.5601
average	25	0.0138	0.1065	0.0512	0.0904	0.4956	0.0004	0.2422
average	27	0.0156	0.0540	0.0406	0.2939	0.2841	0.0008	0.3110
average	30	0.0119	0.0141	0.0133	0.1597	0.4279	0.0009	0.3722
average	32	0.0218	0.0081	0.0004	0.0312	0.1806	0.0001	0.7578
average	36	0.0143	0.1100	0.1149	0.1454	0.4237	0.0011	0.1906
average	39	0.0099	0.2082	0.2319	0.2579	0.1861	0.0006	0.1054
average	43	0.0104	0.0612	0.0581	0.0653	0.3679	0.0002	0.4369
average	54	0.0152	0.0378	0.0126	0.0434	0.3764	0.0004	0.5142
average	55	0.0117	0.2293	0.1197	0.2901	0.2724	0.0021	0.0747

B. Mean categorical mapping for blastocyst rate groups

Group	Sample Size	Adaptor_badquality	rRNA	Mito	Genomic	Bactviral	Repbase	Unmapped
High	13	0.0113	0.0729	0.0493	0.0985	0.3361	0.0004	0.4315
Low	14	0.0126	0.0754	0.0588	0.1662	0.3187	0.0010	0.3673
Average	11	0.0133	0.0959	0.0642	0.1478	0.3122	0.0008	0.3657

%Adaptor_bad Adaptor contamination
Repbase Repetitive elements from diverse eukaryotes, *https://www.girinst.org/repbase/*

Supplementary Table 5: miRNAs associated with overlapping target genes

**Enrichment**	**Target Gene***	**Pattern**	**MiR Target Database**	**MiRNAs***	**Target Depletion**
Enriched in Low Blast Rate	GSE1	Up-down	TargetScan	hsa-miR-181b-5p, hsa-miR-495-3p	Depleted all samples
Enriched in Low Blast Rate	NDRG2	Up-down	TargetScan	hsa-miR-1224-5p, hsa-miR-181b-5p	Depleted all samples
Enriched in Low Blast Rate	**PHC3**	Down-same	TargetScan	hsa-miR-495-3p, **hsa-miR-181b-5p**	Depleted all samples
Enriched in Low Blast Rate	TGFBR3	Down-same	TargetScan	hsa-miR-495-3p, hsa-miR-181b-5p	Depleted all samples
Enriched in Low Blast Rate	CS	Down-same	miRTarBase	hsa-miR-200b-5p, hsa-miR-365b-3p	Depleted all samples
Enriched in Low Blast Rate	MOCS3	Up-down	miRTarBase	hsa-miR-619-5p, hsa-miR-6780a-5p	Depleted all samples
Enriched in Low Blast Rate	NPM1	Up-down	miRTarBase	hsa-miR-296-3p, hsa-miR-92a-3p	Depleted all samples
Enriched in Low Blast Rate	**PHC3**	Down-same	miRTarBase	hsa-miR-296-3p, **hsa-miR-181b-5p**	Depleted all samples
Enriched in Low Blast Rate	VPS53	Down-down	miRTarBase	hsa-miR-619-5p, hsa-miR-6780a-5p	Depleted all samples
Enriched in Low Blast Rate	ZNF675	Up-down	miRTarBase	hsa-miR-365b-3p, hsa-miR-200b-5p	Depleted all samples
Enriched in High Blast Rate	**AKIRIN1**	Down-same	TargetScan	hsa-miR-145-5p, **hsa-miR-224-5p**, hsa-miR-590-5p	Depleted all samples
Enriched in High Blast Rate	ATF2	Up-down	TargetScan	hsa-miR-19a-3p, hsa-miR-224-5p, hsa-miR-485-5p	Depleted all samples
Enriched in High Blast Rate	CIT	Up-down	TargetScan	hsa-miR-19a-3p, hsa-miR-224-5p	Full-length in 1 sample, but depleted in remaining samples
Enriched in High Blast Rate	DLG1	Down-same	TargetScan	hsa-miR-19a-3p, hsa-miR-219a-2-3p	Depleted all samples
Enriched in High Blast Rate	FGF10	Down-same	TargetScan	hsa-miR-145-5p, hsa-miR-19a-3p	Depleted all samples
Enriched in High Blast Rate	MAP3K1	Up-down	TargetScan	hsa-miR-145-5p, hsa-miR-19a-3p, hsa-miR-488-3p, hsa-miR-590-5p	Depleted all samples
Enriched in High Blast Rate	TMEM63B	Down-same	TargetScan	hsa-miR-19a-3p, hsa-miR-145-5p	5’ depleted (all missing first 6 exons)
Enriched in High Blast Rate	TNR	Down-same	TargetScan	hsa-miR-139-5p, hsa-miR-488-3p	Depleted all samples
Enriched in High Blast Rate	**USP37**	Up-down	TargetScan	hsa-miR-145-5p, **hsa-miR-19a-3p**, hsa-miR-224-5p	5’ depleted (all missing at least the first exon)
Enriched in High Blast Rate	ZRANB2	Up-down	TargetScan	hsa-miR-139-5p, hsa-miR-19a-3p, hsa-miR-219a-2-3p	Depleted all samples
Enriched in High Blast Rate	ACTN4	Down-same	miRTarBase	hsa-miR-324-3p, hsa-miR-548o-5p	Depleted all samples
Enriched in High Blast Rate	**AKIRIN1**	Down-same	miRTarBase	**hsa-miR-224-5p**, hsa-miR-7-1-3p	Depleted all samples
Enriched in High Blast Rate	FAXC	Down-same	miRTarBase	hsa-miR-324-3p, hsa-miR-488-3p	Depleted all samples
Enriched in High Blast Rate	PRRC2C	Up-down	miRTarBase	hsa-miR-324-3p, hsa-miR-19a-3p	Depleted all samples
Enriched in High Blast Rate	PTMA	Up-down	miRTarBase	hsa-miR-616-3p, hsa-miR-7-1-3p, hsa-miR-6766-5p	Depleted all samples
Enriched in High Blast Rate	QKI	Up-down	miRTarBase	hsa-miR-19a-3p, hsa-miR-224-5p	Full-length in 9 samples, depleted in remaining samples
Enriched in High Blast Rate	RAB13	Up-down	miRTarBase	hsa-miR-19a-3p, hsa-miR-485-5p	Depleted all samples
Enriched in High Blast Rate	SYNJ2	Up-down	miRTarBase	hsa-miR-324-3p, hsa-miR-485-5p	Depleted all samples
Enriched in High Blast Rate	TPM3	Up-down	miRTarBase	hsa-miR-145-5p, hsa-miR-488-3p, hsa-miR-6766-5p, hsa-miR-485-5p	Depleted all samples
Enriched in High Blast Rate	**USP37**	Up-down	miRTarBase	hsa-miR-616-3p, **hsa-miR-19a-3p**	5’ depleted (all missing at least the first exon)

***Bolding of target genes and miRNAs indicates they are within both miRNA target databases**
